# High prevalence of IgG antibodies to Ebola virus in the Efé pygmy population in the Watsa region, Democratic Republic of the Congo

**DOI:** 10.1186/s12879-016-1607-y

**Published:** 2016-06-10

**Authors:** Sabue Mulangu, Matthias Borchert, Janusz Paweska, Antoine Tshomba, Afongenda Afounde, Amayo Kulidri, Robert Swanepoel, Jean-Jacques Muyembe-Tamfum, Patrick Van der Stuyft

**Affiliations:** National Institute for Biomedical Research, Kinshasa, Democratic Republic of the Congo and, University of Kinshasa, Kinshasa, Democratic Republic of the Congo; Southern African Centre for Infectious Diseases and Surveillance, Morogoro, Tanzania; Institute of Tropical Medicine, Antwerp, Belgium; Current affiliation: Institute of Tropical Medicine and International Health, Charité – Universitätsmedizin Berlin, Berlin, Germany; National Institute for Communicable Diseases, Johannesburg, South Africa; Université de Kisangani, Faculté de Médecine, Département de santé publique, Kisangani, Democratic Republic of the Congo; Ministry of Health, Watsa, Democratic Republic of the Congo; Current affiliation: Department of Veterinary Tropical Diseases, University of Pretoria, Pretoria, South Africa; Ghent University, Ghent, Belgium

**Keywords:** Ebola virus, IgG Antibody, Pygmy

## Abstract

**Background:**

Factors related to the natural transmission of Ebola virus (EBOV) to humans are still not well defined. Results of previous sero-prevalence studies suggest that circulation of EBOV in human population is common in sub-Saharan Africa. The Efé pygmies living in Democratic Republic of the Congo are known to be exposed to potential risk factors of EBOV infection such as bush meat hunting, entry into caves, and contact with bats. We studied the pygmy population of Watsa region to determine seroprevalence to EBOV infection and possible risks factors.

**Method:**

Volunteer participants (*N =* 300) aged 10 years or above were interviewed about behavior that may constitute risk factors for transmission of EBOV, including exposures to rats, bats, monkeys and entry into caves. Samples of venous blood were collected and tested for IgG antibody against EBOV by enzyme-linked immunosorbent assay (ELISA). The χ2-test and Fisher’s exact test were used for the comparison of proportions and the Student’s t-test to compare means. The association between age group and anti-EBOV IgG prevalence was analysed by a nonparametric test for trend.

**Results:**

The prevalence of anti-EBOV IgG was 18.7 % overall and increased significantly with age (*p =* 0.023). No association was observed with exposure to risk factors (contacts with rats, bats, monkeys, or entry into caves).

**Conclusions:**

The seroprevalence of IgG antibody to EBOV in pygmies in Watsa region is among the highest ever reported, but it remains unclear which exposures might lead to this high infection rate calling for further ecological and behavioural studies.

**Electronic supplementary material:**

The online version of this article (doi:10.1186/s12879-016-1607-y) contains supplementary material, which is available to authorized users.

## Background

The *Ebolavirus* genus, which is in the *Filoviridae* family, comprises five distinct virus species: Zaire ebolavirus (EBOV), Sudan ebolavirus (SUDV), Tai Forest ebolavirus (TAFV), Reston ebolavirus (RESTV), and Bundibugyo ebolavirus (BDBV) [1]. EBOV, SUDV, and BDBV have caused outbreaks of Ebola virus disease (EVD) in humans with case fatality proportions ranging from 25 to 90 % depending on species [2].

There is currently no specific treatment or vaccine against EBOV approved for human use. Human-to-human transmission of the virus is controlled by the implementation of strict public health procedures, including isolation of probable or confirmed cases. However, interventions that aim to prevent the primary introduction of EBOV into human populations are difficult to implement since all the determinants of virus spill over from its natural reservoirs into the human population are still unknown.

Until recently, EVD outbreak was considered as an emerging zoonotic viral disease that occurred mainly in rural areas of Central Africa. But in December 2013, an Ebola outbreak has emerged in West Africa which was unprecedented in magnitude and spread [3]. Historically, outbreaks of EVD have occurred in sub-Saharan Africa including in Sudan, Uganda, Gabon, Côte d’Ivoire, Democratic Republic of the Congo (DRC), and the Republic of the Congo. The majority of human outbreaks have been associated with the manipulation of carcasses of infected great apes or other wildlife in Gabon and Republic of the Congo [4]. In other settings where non-human primates (NHP) or other wildlife were not identified as the cause of outbreaks (e.g., in DRC, Uganda, and Sudan), epidemiological investigations based on information available from index cases have suggested that bats might be a source of EVD outbreak [5, 6]. This hypothesis has been supported by the discovery of EBOV viral gene sequences from fruit bat species, and others bats have been found to be seropositive for EBOV antigens [7]. Fruit bats were also suggested as possible original source of infection based on the ecological investigation of the current EVD outbreak in Western African countries [8]. However, it remains unclear how bats or other carriers actually transmit the virus to humans, NHPs, or other non-identified hosts, and the parameters that lead to the occurrence of outbreaks remain to be determined.

Several epidemiological studies have been carried out to determine the prevalence of past EBOV infections and associated risk factors in human populations in countries where EVD outbreaks have occurred (e.g., DRC and Gabon) [9–13] and in countries without a history of epidemics (e.g., Cameroon, Central Africa and Liberia) [14–19]. Together, these studies have demonstrated that, although EVD outbreaks are sporadic, exposure to EBOV or ebola-like virus is not a rare event in human populations in sub-Saharan Africa and that seroprelavence is higher in the rainforest ecosystem and in hunter populations.

Pygmies are hunter-gatherer populations living in tropical rainforests in Africa and are known for their nomadic life style that potentially exposes them to EBOV [20]. Ebola seroprevalence studies have been conducted in pygmy populations living in Central African countries including Gabon, Cameroon, and Central African Republic [10, 14, 15]. In DRC, where at least six documented outbreaks of EVD have occurred in the last two decades, several pygmy groups live in the equatorial rainforests, but there are no published data regarding their exposures to EBOV. In 2002, a Marburg haemorrhagic fever (MHF) seroprevalence survey was conducted in the Efé pygmy population living in the Watsa region in north-eastern DRC, where an MHF outbreak occurred between 1998 and 2001 [21]. The main victims of this MHF outbreak were gold miners from the Gorumbwa mine in Durba village and their family members [22]. There was zero prevalence of IgG anti-Marburg antibody in 300 pygmies [21], even though they were reported to be more exposed to risk factors (e.g., hunting and butchering bush meat, entry into caves, and contacts with bats) associated with filovirus infection [7, 23–25] compared to the general, non-pygmy population.

To explore whether the Efé pygmy lifestyle in the Watsa region is related to exposure to EBOV, we investigated previously collected serum samples for evidence of past EBOV infection and tested associations with potential risk factors.

## Methods

The area, study population, interviews, and blood collections are described elsewhere [21]. In brief, the Watsa region in north-eastern DRC had approximately 180,000 inhabitants including 4,000 pygmies living mainly in the southern part of the region. The study participants came from 39 different settlements and voluntarily reported to study sites for interview. During a three days survey in August 2002, after obtaining informed verbal consent, participants were asked about their subsistence activities and contact with wild animals as potential risk factors of primary filovirus transmission. Exposures to persons presumably sick with haemorrhagic fever (define as severe illness with high fever and bleeding from the nose, mouth, and/or anus) in the hospital and at home, receiving invasive medical or traditional treatment were asked as potential risk factors of secondary filovirus transmission.

We collected a blood sample from each volunteer and stored sera were tested for IgG antibodies to EBOV antigen by enzyme-linked immunosorbent assay (ELISA) at the National Institute for Communicable Diseases, Johannesburg as previously described [26]. Briefly, ELISA plates were coated with EBOV antigens diluted 1:1000 in PBS, overnight at +4 °C. Uninfected Vero cell culture antigens were coated in the same conditions on ELISA plates as controls. Diluted sera 1:400 in 5 % non-fat milk in PBS-Tween 20 (0.1 %) were incubated in the wells overnight at +4 °C. Peroxidase-labelled antibody to human IgG was added to wells and the TMB detector system was used to detect binding. We computed the corrected optical density (OD) as the optical density of the EBOV-coated well subtracted by the OD of the corresponding control well. Corrected ELISA optical density values were expressed as percent positivity (PP) of a human serum sample confirmed positive for EBOV and used as an internal control. Cut-off value for recording positive results were deliberately selected to be stringent at 2 × (mean +3 standard deviations (SD)) PP values determined for 60 serum samples from South African controls subjects who were almost certain to be seronegative.

The interview data were recorded in a database using EpiInfo 6.04 (Centers of Disease Control and Prevention, Atlanta, Georgia, USA), and analysed with STATA 12 (College Station, Texas, USA). When comparing characteristics between seropositive and seronegative individuals the χ^2^-test and Fisher’s exact test were used to compare proportions and the Student t-test was used to compare means. We computed SD for means and confidence intervals (CI) of proportions using the exact method. Odds Ratios (OR) and exact 95 % confidence intervals (CI) were used to access the association between risk factors and EBOV IgG seroprevalence. The association between age group and anti-EBOV IgG prevalence was analysed by a nonparametric Cuzick’s test for trend [27].

The representative of the Ministry of Health in the Watsa region and the ethics committee of the Institute of Tropical Medicine in Antwerp approved the study protocol.

## Results

As previously published, a total of 300 pygmies were enrolled into the study and provided blood samples [21]. The proportion of male and female individuals was comparable. EBOV IgG antibodies were found in 56 of the 300 participants, resulting in an overall prevalence of anti-EBOV IgG in the pygmy population of 18.7 % (95 % CI 14.4–23.5 %): 20 % (95 % CI 13.9–27.3 %) in males and 17 % (95 % CI 11.7–24.4 %) in females. The difference between men and women was not statistically significant in any age group (χ^2^-test *p =* 0.35).

**Fig. 1:**
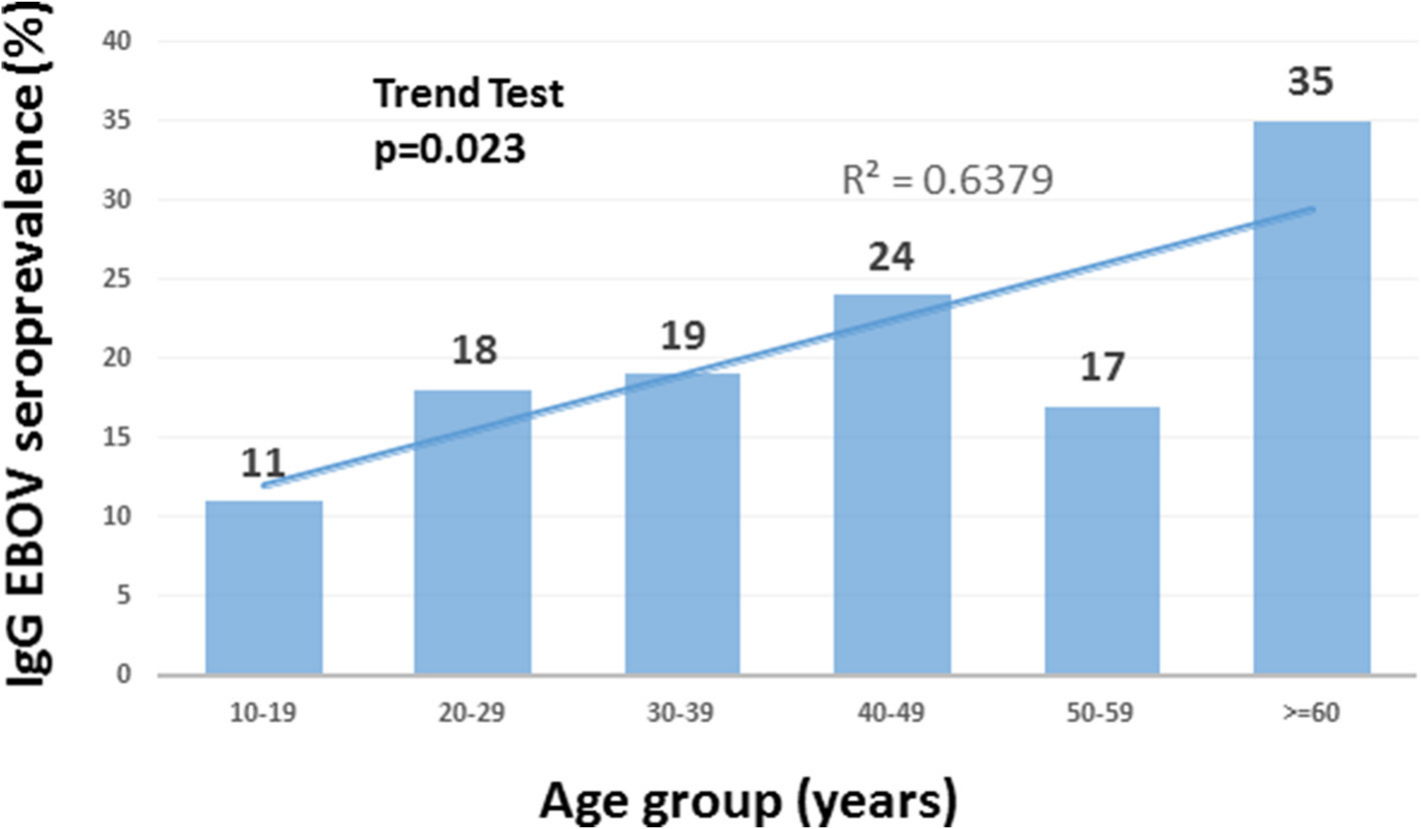
Age distribution of individuals with IgG EBOV antibodies in the Watsa area, DRC (*p =* 0.023)

The mean age of the study participants was 32 years (±SD 14.6). Antibody-positive participants were significantly older than antibody-negative participants (35.9 ± 14.7 vs. 31.3 ± 14.5; Student t-test *p =* 0.03). EBOV antibody-positive participants were seen in all age groups, the youngest IgG EBOV-positive participant being 12 and the oldest 65 years old. A significant (Cuzick’s test *p =* 0.023) linear increase in EBOV IgG prevalence was noted with increasing age group (Fig. 1).

There was no statistically significant difference in EBOV seroprevalence between groups exposed or unexposed to potential risk factors of primary filovirus transmission (hunting, entering caves, and contact with wild animals) or of secondary transmission in households or hospitals (Table 1).

**Table 1:** Antibodies to EBOV and potential risk factors for Ebola virus disease fever in pygmies residing in the Watsa Region, DRC

Potential risk factors: N exposed (% exposed)	Exposed # IgG pos. (%)	Non exposed # IgG pos. (%)	χ2-test p	OR (95 % CI)
Risk factors for primary transmission				
Subsistence activities				
Hunting: 180 (60)	37 (20.6)	19 (15.8)	0.3	1.38 (0.72, 2.68)
Entering caves: 295 (98)	55 (18.6)	1 (20.0)	0.66^b^	1.19 (0.64, 2.23)
Contacts with wild animals				
Rodents				
Touched: 216 (72)	42 (19.4)	14 (6.7)	0.58	1.21 (0.60, 2.54)
eaten^a^ : 127 (42)	20 (15.8)	36 (20.8)	0.27	0.71 (0.37–1.35)
bitten by : 91 (30)	15 (16.5)	41(19.6)	0.52	0.81 (0 .39, 1.60)
any contact : 238 (79)	45 (18.9)	11 (17.7)	0.83	1.08 (0.53, 2.33)
Bats				
Touched : 224 (75)	43 (19.6)	13 (17.1)	0.69	1.15 (0.56, 2.49)
eaten^a^ : 160 (53)	33 (20.6)	23 (16.4)	0.35	1.03 (0 .21, 10.10)
bitten by : 57 (19)	9 (16.1)	47 (19.3)	0.53	0.78 (0.32, 1.76)
any contact : 233 (78)	45 (19.3)	11 (16.4)	0.59	1.21 (0.60, 2.61)
Monkeys, apes				
Touched : 273 (91)	52 (19.0)	4 (14.8)	0.80^b^	1.35 (0 .44, 5.61)
eaten^a^ : 289 (96)	54 (18.7)	2 (18.2)	1.00^b^	1.03 (0 .21, 10.10)
bitten by : 16 (5)	3 (18.8)	53 (18.7)	1.00^b^	1.01 (0 .18, 3.84)
any contact : 294 (98)	56 (19.0)	0 (0.0)	0.28^b^	
Wild animals: any contact : 296 (97)	56 (18.9)	0 (0.0)	0.43^b^	
Risk factors for secondary transmission				
Contact with someone suffering from HF^§^				
had someone with HF in the household : 66 (22)	7 (10.6)	49 (20.9)	0.06	0.45 (0.16, 1.07)
been in the same room with someone with HF : 47 (16)	8 (17.0)	48 (18.9)	0.75	0.88 (0.33, 2.06)
worked with someone with HF : 61 (20)	9 (14.7)	47 (19.7)	0.38	0.71 (0.29, 1.59)
participated in funeral of someone with HF : 65 (22)	12 (18.5)	44 (18.7)	0.96	0.98 (0.44, 2.07)
touched someone with HF : 57 (19)	11 (19.3)	45 (18.5)	0.89	1.05 (0.45, 2.27)
touched blood, urine, faeces of someone with HF : 34 (11)	8 (23.5)	48 (18.0)	0.44	1.40 (0.51, 3.42)
touched remains of someone with HF : 44 (15)	12 (27.3)	44 (17.2)	0.11	1.81 (0.78, 3.94)
any contact : 102 (34)	16 (15.7)	40 (20.2)	0.34	0.74 (0.38, 1.38)
any direct contact (touched) : 79 (26)	16 (20.3)	40 (18.1)	0.67	1.15 (0.59, 2.18)
Invasive medical treatment				
ever received injection : 263 (88)	51 (19.4)	5 (13.5)	0.39	1.54 (0.56, 5.31)
ever received surgical or obstetric care : 124 (41)	27 (21.8)	29 (16.5)	0.24	1.41 (0 .75, 2.63)
any invasive medical treatment ever : 278 (93)	54 (19.4)	2 (9.1)	0.18^b^	2.41 (0.63, 15.66)
Traditional treatment				
ever had scarification : 294 (98)	54 (18.4)	2 (33.3)	0.4	0 .45 (0.06, 5.11)

^a^Bush meat often is smoked, grilled or cooked; exposure to viable virus may therefore be more likely to happen during preparation of such meat for consumption than during consumption itself

^b^Fisher’s Exact Test

§: HF (haemorrhagic fever): severe illness with high fever and bleeding from the nose, mouth, and/or anus

The IgG EBOV prevalence was higher in study participants who had experienced a febrile haemorrhagic syndrome at least once in their life than those who had not, but the difference was not statistically significant (22 % vs. 16 %; χ^2^-test *p =* 0.18).

## Discussion

Here, we examined the seroprevalence of EBOV in a pygmy population in the Watsa area of DRC to establish whether their previously reported increased exposure to risk factors than the general population was associated with filovirus infection.

There was a very high EBOV antibody prevalence (18.7 %) in this study population, consistent with previous studies of pygmy populations in Cameroon (14.5 %) [15], Central African Republic (7.0 %), [14] and Gabon (11.1 %) [10]. Ebola ELISA was used as the serological test in the studies in Central African Republic and Gabon, whereas an immunofluorescence antibody (IFA) test was used in the Cameroon study. The ELISA test we used had Zaire ebolavirus species as source of antigen. This assay is more specific and sensitive for the detection of past infection than the standard IFA test [28, 29] and has the potential to detect cross-species ebolavirus antibodies [30]. Therefore, the pygmy seroprevalence reflects a relatively high level of exposure to EBOV or to other cross-reactive ebolavirus species in the study area. Since the EBOV species is known to be very pathogenic with a high case fatality proportion, this seroprevalence might underestimate the level of exposure to EBOV by assessing only the survivors. Alternately, our study population may have been exposed to other known or unknown ebolavirus species of lower pathogenicity, which may cause cross-reaction with the assay and contribute to the high seroprevalence [30, 31].

This study was a retrospective survey using stored pygmy sera, and comparison sera from the general sedentary population in the Watsa area were unavailable. However, two EBOV seroprevalence studies using the same ELISA assay conducted in the sedentary population in Kikwit city (the epicenter of a major EVD outbreak in 1995) and neighbouring villages, DRC, reported a prevalence of 2.2 % and 9.3 %, respectively [9]. The fact that the Efé pygmy population in Watsa tended to have a higher EBOV prevalence than non-pygmy inhabitants of Kikwit city and surrounding villages is in line with studies in Cameroon, Gabon, and Centre African Republic [10, 14, 15].

We found that EBOV IgG seroprevalence in Watsa pygmies increases significantly with age and is maximal in the 60 years and older age group. Other EBOV seroprevalence studies have shown that seroprevalence was highest in individuals aged between 20 and 40 years [9, 10, 14, 15]. The different distributions of seroprevalence by age may be due to differences in lifestyle between study populations. Non-pygmies may be more likely to enter deep into the rain forest for economic survival activities and to be exposed to a variety of putative EBOV animal hosts when they are in the economically most active 20–40 year age group. The Efé pygmies, however, who live in the rain forest all their life, may accumulate exposure to putative EBOV animal hosts throughout life, which is reflected by a linear seroprevalence increase with age. The fact that EBOV seroprevalence in pygmies in RCA [14] and Gabon [10] was highest in the 20-40 year old age group may reflect the trend that Aka and Baka pygmies in those areas have started to abandon their traditional semi-nomadic lifestyle in favour of the sedentary farmer’s life [32].

Here, there was no association between risk factors for primary or secondary transmission of filoviruses and EBOV antibody status. Some of the non-significant differences observed may be real, but our study may have been insufficiently powered to detect them. For instance, categorisation of the level of exposure for participants in contact with someone suffering from haemorrhagic fever might reveal significant differences. Furthermore, our study was limited by the fact that study participants were not selected randomly but were self-recruited, although we have no reason to believe that this resulted in selection bias.

## Conclusion

Pygmy populations are known to be particularly at risk of filovirus exposure compared to sedentary populations. Here, pygmies in the Watsa region of DRC were at high risk and had one of the highest reported seroprevalence of EBOV IgG antibodies using a well-established ELISA assay. Since we could not identify risk factors that could explain this high prevalence, further ecological and behavioural studies might be necessary to better understand determinants of EBOV exposure in this population.

## Abbreviations

BDBV: bundibugyo ebolavirus, CI: confidence intervals, DRC: Democratic Republic of the Congo, EBOV: zaire ebolavirus, ELISA: enzyme-linked immunosorbent assay, EVD: ebola virus disease, IFA: immunofluorescence antibody, MHF: marburg Hemorrhagic Fever, NHP: non-human primates, OD: optical density, OR: odds ratios, PP: percent positivity, RESTV: reston ebolavirus, SD: standard deviations, SUDV: sudan ebolavirus, TAFV: tai forest ebolavirus

## Additional file

Additional file 1: Efe pygmy, Watsa, DRC: EBOV exposures data. (XLSX 49 kb)
